# Complete Organelle Genome of the Desiccation-Tolerant (DT) Moss *Tortula atrovirens* and Comparative Analysis of the Pottiaceae Family

**DOI:** 10.3390/genes15060782

**Published:** 2024-06-13

**Authors:** Yang Ma, Lifang Zhang, Min Yang, Qin Qi, Qian Yang, Jordi López-Pujol, Lihong Wang, Dongping Zhao

**Affiliations:** 1 School of Life Sciences, Inner Mongolia University, Hohhot 010070, China; mayang0124@126.com (Y.M.);; 2 School of Ecology and Environment, Inner Mongolia University, Hohhot 010020, China; 3 Botanic Institute of Barcelona (IBB), CSIC-CMCNB, 08038 Barcelona, Spain; jlopez@ibb.csic.es; 4 Escuela de Ciencias Ambientales, Universidad Espíritu Santo (UEES), Samborondón 091650, Ecuador; 5 Key Laboratory of Herbage & Endemic Crop Biotechnology, Ministry of Education, School of Life Sciences, Inner Mongolia University, Hohhot 010070, China

**Keywords:** volcano, biological soil crust, bryophyte, chloroplast genome, mitochondrial genome

## Abstract

*Tortula atrovirens* (Sm.) Lindb. is an important component of biological soil crusts and possesses an extraordinary tolerance against desiccation in dryland habitats. However, knowledge of the organelle genome of this desiccation-tolerant (DT) moss is still lacking. Here, we assembled the first reported *Tortula* organelle genome and conducted a comprehensive analysis within the Pottiaceae family. *T. atrovirens* exhibited the second largest chloroplast genome (129,646 bp) within the Pottiaceae, whereas its mitogenome (105,877 bp) and those of other mosses were smaller in size compared to other land plants. The chloroplast and mitochondrial genomes of *T. atrovirens* were characterized by the expansion of IR boundaries and the absence of homologous recombination-mediated by large repeats. A total of 57 RNA editing sites were detected through mapping RNA-seq data. Moreover, the gene content and order were highly conserved among the Pottiaceae organelle genomes. Phylogenetic analysis showed that bryophytes are paraphyletic, with their three lineages (hornworts, mosses, and liverworts) and vascular plants forming successive sister clades. *Timmiella anomala* is clearly separated from the monophyletic Pottiaceae, and *T. atrovirens* is closely related to *Syntrichia filaris* within the Pottioideae. In addition, we detected four hypervariable regions for candidate-molecular markers. Our findings provide valuable insights into the organelle genomes of *T. atrovirens* and the evolutionary relationships within the Pottiaceae family, facilitating future discovery of DT genetic resources from bryophytes.

## 1. Introduction

Plastids and mitochondria are crucial semi-autonomous organelles that possess genomes of endosymbiotic origin and exhibit uniparental inheritance [1]. Recent advances in sequencing technologies have boosted the publication of organelle genomes. As of September 2023, the NCBI database has released approximately 13,000 plastomes and 673 mitogenomes, but only 285 species have assembled both genomes [2]. Despite the fact that divergent evolutionary trajectories have been proposed for them, there are still some knowledge gaps, mostly related to unequal sampling. Bryophytes comprise ca. 20,000 known species (second only to angiosperms in species richness among land plants) that exhibit remarkable eco-physiological advantages for adapting to modern ecosystems in almost all terrestrial habitats [3]. Unfortunately, only 85 plastomes and 190 mitogenomes of bryophytes are reported to date, in sharp contrast to 12,142 plastomes and 253 mitogenomes available for angiosperms [2]. Previous studies have indicated that the plastomes of bryophytes display the same well-conserved plastome structures for angiosperms [4]; in contrast, their mitogenomes show conserved structural evolution within the three bryophyte lineages (hornworts, mosses, and liverworts), a somewhat different picture to the highly variable mitogenomes of angiosperms [5]. However, understanding the organelle genome information for bryophytes is presently limited.

The family Pottiaceae is the most species-rich moss family and consists of around 1425 species from 90 genera, representing over 10% of the 10,000 to 15,000 known moss species [3,6]. This family is one of the representatives of desiccation-tolerant (DT) lineages and typically thrives in harsh environments, often dominating the ground vegetation of arid regions throughout the world [7,8]. The taxonomy and phylogeny of a few major genera, such as *Oxystegus* [9], *Pseudocrossidium* [10], and *Didymodon* [11], have been demonstrated complex through the application of morphological and molecular analysis. Nevertheless, its generic circumscription and topological relationships within the family remain largely unexplored using molecular data [11], particularly in the absence of organelle genome sequencing. Currently, the complete organelle genome of both chloroplast and mitochondrion has been documented for only eight species from different genera, and the systematic comparative analysis of these genomes is yet to be carried out. The scarcity of resources impedes our comprehension of the evolutionary dynamics of the Pottiaceae organelle genomes.

The generic boundaries of genus *Tortula* Hedw. and its relationships with *Pottia*, *Phascum*, *Desmatodon*, *Chenia*, *Dolotortula*, *Hennediella*, *Hilpertia*, *Sagenotortula*, *Stonea*, and *Syntrichia*, are unclear and remain the subject of ongoing debate [7,12]. *Tortula atrovirens* (Sm.) Lindb. is a DT moss with a widespread distribution in temperate regions, generally found on calcareous exposed or rocky soils, or on volcanic ashes. It is an important component of biological soil crusts and has received considerable attention for its remarkable tolerance to desiccation in northern China [13]. Ultrastructural observations indicate that the membranes of vegetative cells in DT mosses do not exhibit observable damage during desiccation [14]. Thylakoids, grana, and mitochondrial cristae remain intact throughout the drying and re-wetting cycle [15]. However, there are significant changes in the form of the organelles, particularly the chloroplasts, which have prominent lobes and lamellar extensions in the hydrated state but become rounded when desiccated, gradually returning to their normal state within approximately 24 h after rehydration (R24) [15]. The most over-represented upregulated R24 GO categories were “plastid”, “thylakoid”, and “photosynthesis” in *Bryum argenteum* [16]. Previous studies have documented several unique morphological characteristics related to DT in *T. atrovirens*. Specifically, leaves become contorted-curved when dry, but spread when moist; in addition, leaf margins are strongly recurved, the ventral side of the upper costa is covered by a pad of inflated, papillose cells, and each upper leaf cell is covered by 2–4 C-shaped papillae. However, there is a lack of studies using the organelle genome of *Tottula* lineage to understand its desiccation stress response and resolve the phylogenetic position of *T. atrovirens*.

Here, we assembled the high-quality organelle genome of *T. atrovirens* by utilizing a hybrid approach combining PacBio-HiFi and Illumina sequencing strategies, and performed a comprehensive analysis among different species of Pottiaceae. Our study aimed to (1) characterize the organelle genome of *T. atrovirens*, (2) explore the evolutionary relationships of Pottiaceae, and (3) develop the hypervariable regions as candidate DNA barcoding to distinguish Pottiaceae species. These findings will expand genetic information available for studying the phylogeny and evolution of Pottiaceae and facilitate further utilization of DT genetic resources.

## 2. Materials and Methods

### 2.1. Sampling and DNA/RNA Extraction

**Figure 1:**
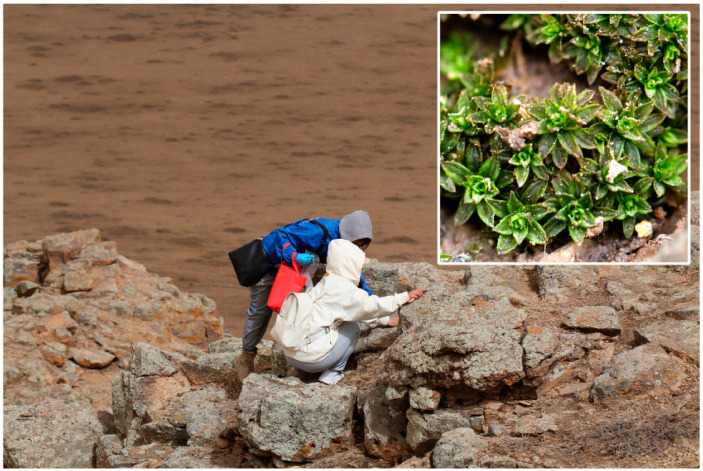
Sampling *Tortula atrovirens* from the Ulanhada Volcano in China.

Samples of *T. atrovirens* (Figure 1) were collected from the Ulanhada Volcano in Ulanqab, Inner Mongolia Autonomous Region, China (113°5′08.23″ E, 41°33′12.67″ N). The voucher specimen was deposited in the Herbarium of Inner Mongolia University. Genomic DNA and total RNA were extracted from the axenic in vitro cultivation of gametophytes isolated from a single sample [17] using the modified CTAB method [18] and RNeasy Plus Mini Kit (Qiagen Inc., Hilden, Germany).

### 2.2. Library Construction and Sequencing

A high-quality organelle genome was obtained by the combination of PacBio-HiFi and Illumina sequencing strategies. Paired-end libraries (350 bp) were prepared according to the manufacturer’s instructions and sequenced on the Illumina HiSeq 2500 System (Illumina Inc., San Diego, CA, USA). SMRTbell libraries (10 to 50 kb) were constructed using SMRTbell Express Template Prep Kit 2.0 following the manufacturer’s protocols and sequenced on a single-molecule real-time (SMRT) long-read sequencing system (Pacific Biosciences, Menlo Park, CA, USA). RNA-seq libraries (350 bp) were prepared with the Truseq RNA Library Prep kit following the manufacturer’s recommendations and sequenced on the Illumina HiSeq 2500 platform (Illumina Inc., San Diego, CA, USA).

### 2.3. Assembly and Annotation

The assembly of chloroplast and mitochondrial genomes were performed using GetOrganelle v1.7.5 [19] and Wtdbg2 v2.5 [20], respectively. To polish the contigs, we employed a hybrid error correction method, including Pilon v1.23 [21] to assemble HiFi long reads and Racon v1.4.20 [22] to assemble Illumina short reads. The annotations were performed on GeSeq v1.43 [23] and manually corrected using Apollo v1.11.8 [24]. Circular maps were drawn by OGDRAW v1.3.1 [25]. PhyloSuite v1.2.1 was used to investigate chloroplast and mitochondrial genome sizes, GC contents, and protein-coding gene (PCG) numbers, respectively [26]. Heatmaps of PCGs were drawn by Tbtools-II v1.120 [27].

### 2.4. IR Boundaries Analysis and Selection of Candidate DNA Barcodes

The inverted repeat (IR) boundaries were visualized in the online IRscope program [28]. The Shuffle-LAGAN mode of mVISTA [29] was employed to perform the structural comparison of eight Pottiaceae chloroplast genomes, using the model moss *Physcomitrella patens* as a reference. Hypervariable regions of the chloroplast genomes were detected by conducting sliding window analysis in DnaSP v5.10 [30]. 

### 2.5. Repetitive Sequences Analysis 

Simple sequence repeats (SSR) were detected by the MISA web server [31] using the same parameters as Asaf et al. [4]: (1/10) (2/8) (3/4) (4/4) (5/3) (6/3). Tandem repeats and dispersed repeats were identified using the TRF [32] and REPuter [33] web servers, respectively. Tbtools-II v1.120 [27] was applied to the visualization of these repetitive sequences. BLASTN was used to identify potential homologous recombination mediated by repeats longer than 50 bp and identity above 85% in the mitogenome.

### 2.6. Codon Usage Preference and RNA Editing Sites Analysis 

PhyloSuite v1.2.2 was used to extract PCGs [26]. MEGA v11.0.11 was applied to analyze codon usage bias and calculate the frequency of relative synonymous codon usage (RSCU) [34]. The RNA-seq data were mapped to organelle genomes using BWA v0.7.10-r789 [35]. The putative RNA editing sites in the PCGs were detected using Samtools v1.7 with depth > 10X [36].

### 2.7. Mitochondrial Plastid Sequences Analysis 

The mitochondrial plastid sequences (MTPTs) were identified by mapping the *T. atrovirens* chloroplast genome against its mitogenome in BLASTN v2.2.25 [37]. Due to the loss of genes observed in the chloroplast genome, the BLASTN analysis was also conducted between the *T. atrovirens* mitogenome and the chloroplast genome of *Takakia lepidozioides*. Circular maps were generated by Tbtools-II v1.120 [27]. 

### 2.8. Synteny and Selective Pressure Analysis

Colinear blocks (longer than 500 bp in size) were identified using BLASTN v2.2.25 [37]. The results of synteny analysis were visualized by MCscanX v0.8 [38] of TBtools [39]. Tbtools-II v1.120 was used to calculate the nonsynonymous substitution rates (*Ka*) and synonymous substitution rates (*Ks*) of PCGs common to the chloroplast and mitochondrial genome, respectively [27]. Heatmaps and Boxplots of *Ka*/*Ks* were generated by Tbtools-II v1.120 [27] and ggplot2 package in R software v4.0.5 [40], respectively.

### 2.9. Phylogenetic Analyses

To infer phylogenetic relationships of Pottiaceae, the chloroplast and mitochondrial genomes of 27 representative species from major lineages of bryophytes were selected and downloaded from NCBI, using two algae (*Mesostigma viride* and *Chara vulgaris*) and two tracheophytes (*Ginkgo biloba* and *Nicotiana tabacum*) as outgroups. Phylogenetic trees were generated using the following six datasets: (1) 62 chloroplast genes under 31 partitioning schemes, (2) an unpartitioned analysis of concatenated first and second codon positions of 62 chloroplast genes, (3) an unpartitioned analysis of concatenated third codon positions of 62 chloroplast genes, (4) 15 mitochondrial genes under 10 partitioning schemes, (5) an unpartitioned analysis of concatenated first and second codon positions of 15 mitochondrial genes, and (6) an unpartitioned analysis of concatenated third codon positions of 15 mitochondrial genes. The shared PCGs were extracted using Phylosuite v1.2.2 [26]. MAFFT v7.450 was used to align the sequences [41]. PartitionFinder2 [42] was used to select the optimal partitioning schemes and their evolutionary models with the following parameters: “linked for branchlengths” and “AICc for the model selection criterion” (Table S1). Maximum likelihood (ML) methods were implemented using IQ-TREE 2.1.4 [43] for concatenated coding sequences. The ML phylogenetic trees were visualized with iTOL v6 [44].

## 3. Results

### 3.1. Feature of Organelle Genome

**Figure 2:**
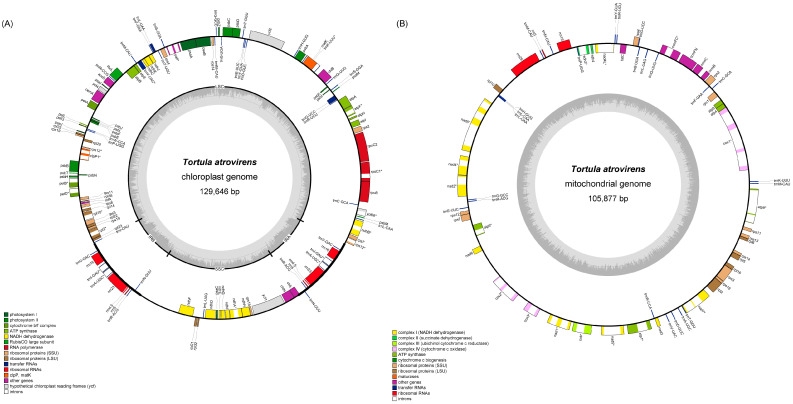
Circular genome maps of the plastome (**A**) and mitogenome (**B**) of *T. atrovirens*. The asterisk indicate intron-containing genes.

The chloroplast genome of *T. atrovirens* was 129,646 bp in length and possessed a circular DNA molecule with the typical quadripartite structure, consisting of a large single-copy (LSC) region of 84,738 bp, a small single-copy (SSC) region of 24,352 bp, and a pair of inverted repeats (IRs) of 10,278 bp (Figure 2; Table S2). Nucleotide composition was 35.87% A, 14.1% T, 14.25% G, and 35.78% C, with an overall GC content of 28.35% (Table S2). It was observed to have lost 12 protein-coding genes (PCGs), namely *ccsA*, *cysA*, *cysT*, *pdf1*, *petN*, *psb30*, *rpoA*, *rps16*, *tufA*, *ycf10*, *ycf12*, and *ycf3*, whereas 125 genes were still intact, including 81 PCGs, 36 tRNA genes, and eight rRNA genes (Figure S1A; Table S3). Among these genes, 15 intron-containing genes were found, 13 of which displayed one intron; only the genes *clpP1* and *rps12* harbored two introns (Table S3).

The mitogenome of *T. atrovirens* assembled as a single circular chromosome of 105,877 bp with 39.63% GC content (Figure 2; Table S2). The mitogenome exhibited a reduction of PCGs, including *matR*, *mttB*, *rps8*, and *rps10* (Figure S1B; Table S4). Nevertheless, it still contained 66 genes, consisting of 39 PCGs, 24 tRNA genes, and three rRNA genes. There were a total of 26 introns distributed over 17 genes (Table S4), among which the gene *cox*1 had the largest number of introns (four).

### 3.2. IR Boundaries Analysis

**Figure 3:**
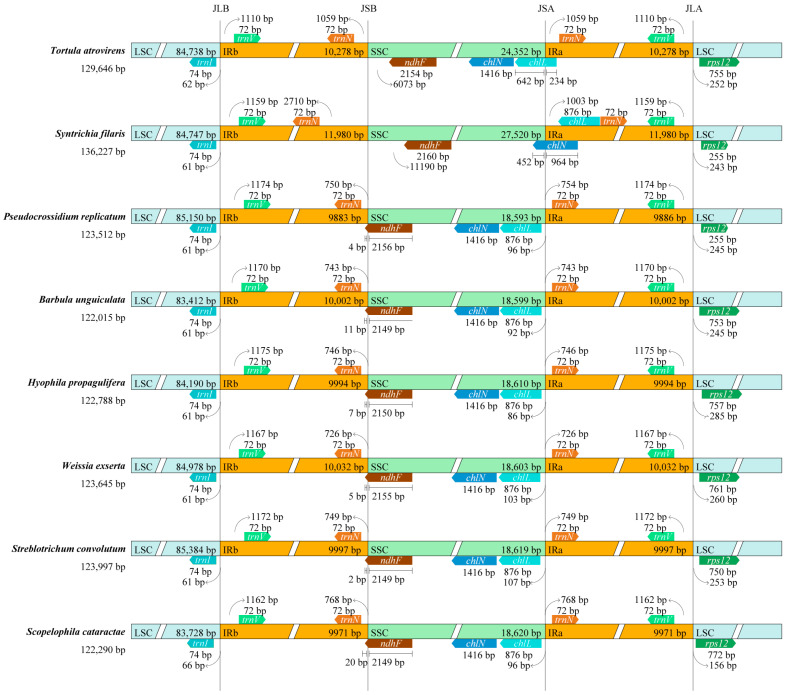
Comparison of IR boundaries among eight Pottiaceae chloroplast genomes.

Significant differences were detected in the length of the IR region across the chloroplast genomes of eight Pottiaceae species, ranging from 19,766 to 23,960 bp (Figure 3). The results revealed that the LSC/IR junctions were highly conserved, whereas the SSC/IR junctions exhibited substantial variability. Specifically, two copies of the *trnN* gene were entirely located in the IR region with different degree of expansion. The distance of *T. atrovirens* and *S. filaris* from the SSC/IR junctions were 1059 and 2710 bp, respectively; those of the remaining species were 726–768 bp. The JSB (IRb/SSC) was located within the *ndhF* gene in all species except for *T. atrovirens* and *S. filaris.* Most part of the *ndhF* gene was located in the SSC region, with only 2–20 bp extending into the IRb region. The *ndhF* gene of *T. atrovirens* and *S. filaris* was completely located in the SSC region, with 6073 and 11,190 bp away from the boundary. The JSA (SSC/IRa) was located within the *chIN* gene in *S. filaris* and the *chIL* gene in *T. atrovirens*, respectively. The *chIN* gene was completely located in the SSC region except for *S. filaris* (in *S. filaris*, 452 bp of the *chIN* gene was located in the SSC region while 964 bp was in the IRa region). Notably, the position of the *chIL* gene varied greatly: in *T. atrovirens*, it spanned the SSC/IRa boundary, that of *S. filaris* was entirely located in the IRa region, and those of the remaining species were entirely located in the SSC region.

### 3.3. Selection of Candidate DNA Barcodes

**Figure 4:**
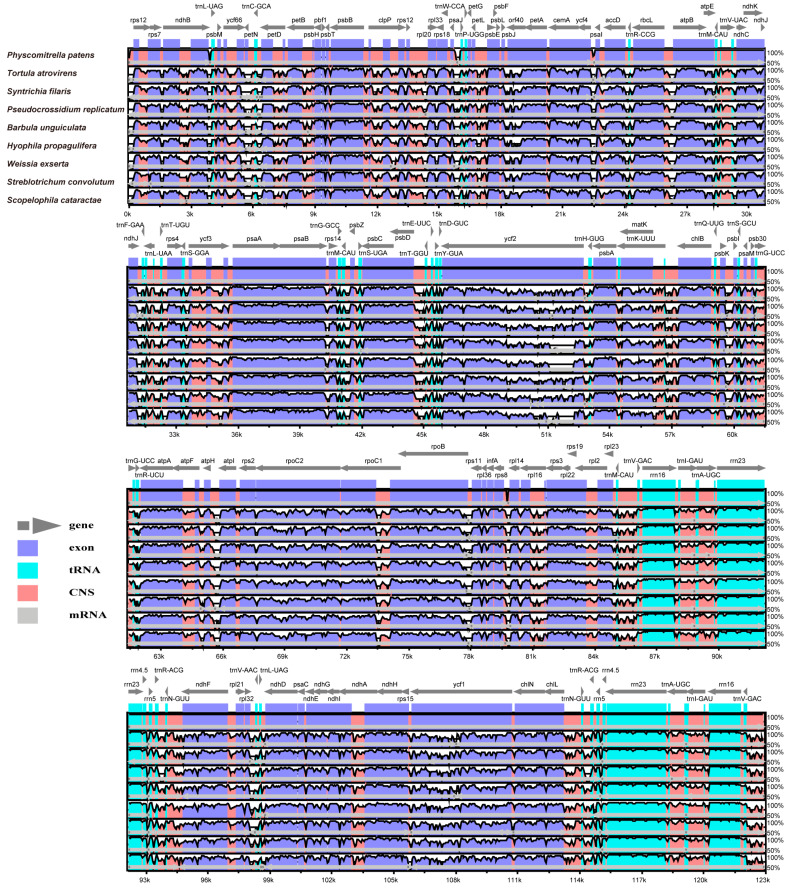
Sequence alignment of eight Pottiaceae chloroplast genomes.

The high level of conservation was also observed in these eight chloroplast genome sequences, with coding regions exhibiting more variability compared to non-coding regions (Figure 4). In addition, we found that SSC and LSC regions exhibited higher divergence than IR regions and identified several significantly differentiated regions, such as *ndhA*-*ndhH*, *ycf1*-c*hIN*, *ndhF*, and *ndhI*-*ndhA* (Figure S3). 

### 3.4. Comparative Analysis of Organelle Genomes

**Figure 5:**
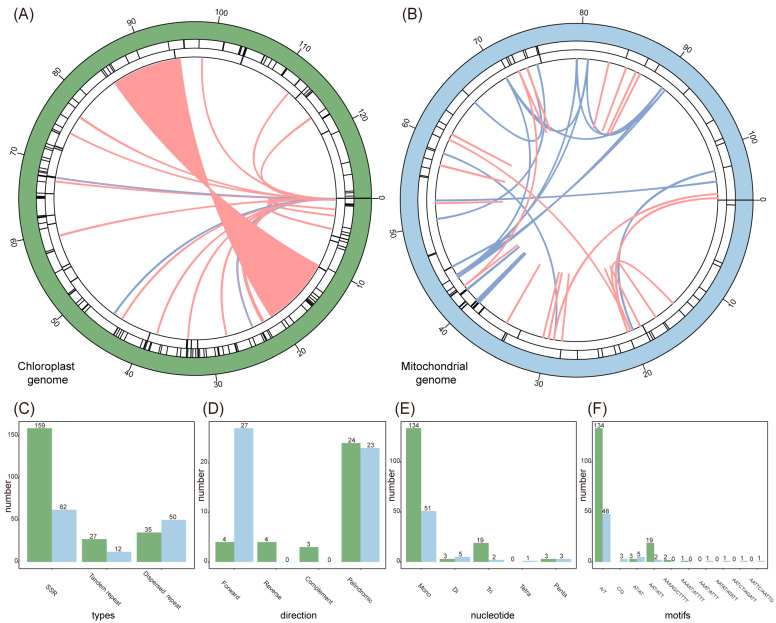
Repetitive sequences of chloroplast (left, green) and mitochondrial (right, blue) genome in *T. atrovirens*. (**A**) Distribution of repeat sequences in chloroplast genome. (**B**) Distribution of repeat sequences in mitochondrial genome. (**C**) Repeat types. (**D**) Dispersed repeat types. (**E**) SSR types. (**F**) SSR motif types. Line colors of blue and pink correspond to forward repeats and palindromic repeats, respectively.

A total of 159 (62) SSRs, 27 (12) tandem repeats, and 35 (50) dispersed repeats were identified in the *T. atrovirens* chl- (mito-) genome, respectively (Figure 5; Tables S5–S10). Furthermore, a total of 4, 4, 3, and 24 forward, reverse, complement, and palindromic repeats were detected in the chloroplast genome, respectively. In contrast, the mitogenome contained 27 forward repeats and 23 palindromic repeats. Monomeric SSRs were the most abundant type, accounting for 84.28% and 82.26% of the total SSRs in the chloroplast and mitochondrial genomes, respectively. The most abundant mono- to tri-repeats in both organelle genomes were A/T (134, 48), AT/AT (3, 5), and AAT/ATT (19, 2). Notably, we only found nine pairs of repeats (>50 bp in size; >85% BLASTN identity) in the mitogenome, with the longest repeat spanning 84 bp (Table S11).

As shown in Figure S3, the 81 chloroplast PCGs comprised 22,596 codons (Table S12), while the 39 mitochondrial PCGs encoded 10,331 codons (Table S13). General preference for relative synonymous codon usage (RSCU) was observed in both organelle genomes, except for the initial codon methionine (Met) and tryptophan (Trp). Additionally, leucine (Leu) emerged as the most prevalent amino acid, whereas cysteine (Cys) was the least abundant.

The chloroplast genome of *T. atrovirens* contained only six RNA editing sites, with the *nad4* gene was edited four times (Table S14). In contrast, the mitogenome of *T. atrovirens* exhibited 51 RNA editing sites, making the C-to-U editing the predominant (accounting for 21.57%; Table S15). The *atp1* gene occurred 30 times, being the most abundant of the PCGs. All the chloroplast sites and most of the mitochondrial sites were at the third position of the triplet codon. 

A total of four homologous fragments were detected in the organelle genome of *T. atrovirens*; with a combined size of 1994 bp, they accounted for 1.54% and 1.88% of the chloroplast and mitochondrial genome, respectively (Figure S4; Table S16). Further annotation results indicated they were two partial rRNA genes (*rrn16S* and *rrn23S*). The homologous fragments were further annotated as partial PCGs (such as *ycf2*, *psbB*, and *rpl22*) and intergenic spacer (IGS) regions. The same results were found from the BLASTN search of *T. atrovirens* mitogenome against the chloroplast genome of *T. lepidozioides* (Table S17).

### 3.5. Synteny and Selective Pressure Analysis

**Figure 6:**
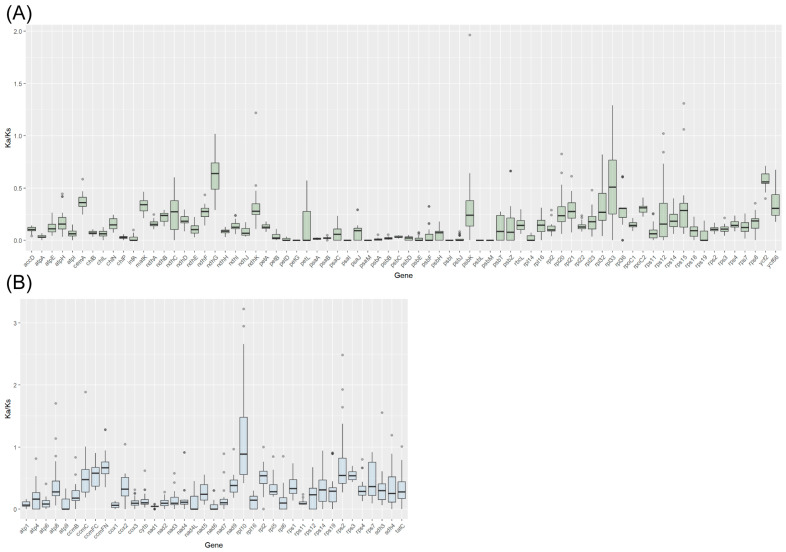
Boxplots of chloroplast (**A**) and mitochondrial (**B**) pairwise *Ka*/*Ks* values among the eight Pottiaceae species.

Based on sequence similarity, we further analyzed the collinearity in the eight Pottiaceae organelle genomes (Figure S5). The results revealed that both exhibited high levels of synteny and consistent arrangement order, displaying structural similarity. A total of 115 PCGs were extracted among the organelle genomes of the eight Pottiaceae species into a super-matrix. Moreover, we calculated the pairwise *Ka*/*Ks* values of these shared PCGs (Figure 6 and Figure S6; Tables S18 and S19). Most *Ka*/*Ks* values of these Pottiaceae species were below 1, indicating that these organelle genes experienced purifying pressure. Notably, the *rpl10* gene showed that most *Ka*/*Ks* ratios were around 1.0, thus suggesting possible positive pressure.

### 3.6. Phylogenetic Analyses

**Figure 7:**
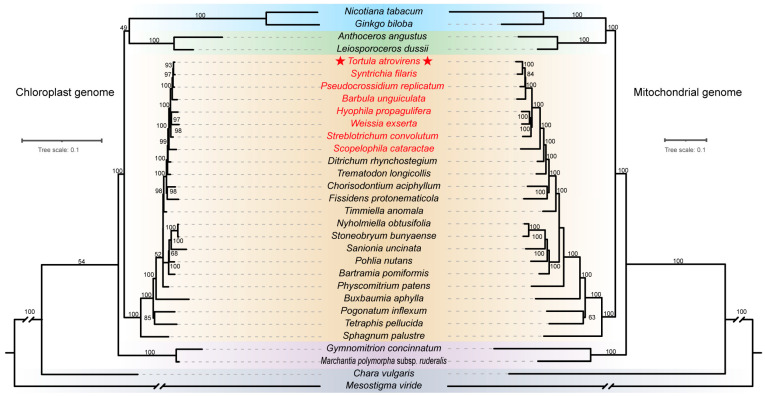
Phylogenetic relationship of *T. atrovirens* with other representative species from major lineages of bryophytes. ML tree based on 62 shared chloroplast genes under 31 partitioning schemes (left) and 15 shared mitochondrial genes under 10 partitioning schemes (right). Numbers above the branches indicate bootstrap values (BS). Background colors in blue, green, yellow, purple, and gray indicate tracheophytes, hornworts, mosses, liverworts, and algae, respectively. Names in red represent the eight Pottiaceae species. Stars (★) indicate the newly sequenced *T. atrovirens*.

Based on 62 shared chloroplast genes and 15 shared mitochondrial genes, maximum likelihood (ML) phylogenetic trees of 31 species (Table S20) were inferred. Four out of six datasets yielded nearly identical topologies with high supporting values (Figure 7 and Figures S7 and S9), with the exception of the two datasets using the concatenated codon positions of 15 mitochondrial genes (Figures S8 and S10). The results showed that bryophytes were paraphyletic. Algaes, liverworts, mosses, and hornworts were successive sister lineages, and hornworts were the closest relatives to tracheophytes. Notably, *T. anomala* that had been formerly placed in Pottiaceae was segregated from this family and resolved at the base of the order Dicranales. The remaining Pottiaceae species formed a monophyletic group, of which the newly sequenced *T. atrovirens* is closely related to *S. filaris*.

## 4. Discussion

### 4.1. Characterization of T. atrovirens Chloroplast Genome 

The expansion and contraction of IR boundaries is thought to be a key factor influencing the size variation of chloroplast genomes [45]. The results of IR boundaries analysis revealed that the IR region of *T. atrovirens* (20,556 bp) and *S. filaris* (23,960 bp) were significantly bigger than those of the remaining six Pottiaceae species (19,766–20,064 bp). The LSC/IR junctions were highly conserved, whereas the SSC/IR junctions exhibited substantial variability, involving the *trnN*, *ndhF*, *chIN*, and *chIL* genes. This could be the reason why *T. atrovirens* and *S. filaris* possess the second 129,646 bp) and the first (136,227 bp) largest chloroplast genomes among the eight studied Pottiaceae species. Moreover, the chloroplast genome of *T. atrovirens* and other Pottiaceae species exhibited highly conserved collinearity and consistent gene order, in common with those of *Tetraphis pellucida* [46] and *T. lepidozioides* [47]. Compared to the model moss *P. patens*, these species lack an inversion with 71 kb in size [48]. In addition, the chloroplast genome of *T. atrovirens* was observed to have lost 12 PCGs; among these, eight genes (*ccsA*, *cysA*, *cysT*, *petN*, *rpoA*, *rps16*, *tufA*, *ycf10*) are absent from nearly all mosses (with the exception of *T. lepidozioides* [4,47]). Notably, the chloroplast genome of *T. atrovirens* lacks the *petN* gene, which presumably has moved into the nuclear genome due to its crucial involvement in photosynthetic electron transport and its relevance to desiccation tolerance [49].

### 4.2. Characterization of T. atrovirens Mitochondrial Genome 

Angiosperm mitogenomes are highly variable in size, ranging from 66 Kb [50] to 11.3 Mb [51]. However, the size comparison of the newly sequenced *T. atrovirens* mitogenome (105,877 bp) with published data evidences that the mitogenomes of mosses (mean: 106 Kb) display rather narrow size variation (99–141 Kb) and are smaller than those of hornworts (mean: 227 Kb) and liverworts (mean: 174 Kb). These findings align with prior research on mitogenomes of the three bryophyte lineages [5]. Unlike the highly dynamic evolution of angiosperms, the structure of bryophyte mitogenomes is generally stable, with the exception of a few case reports, such as one inversion event (ca. 94 kb) in the *Dumortiera hirsuta* (Marchantiales) mitogenome [52] and two rearrangement events of the *Gymnomitrion concinnatum* (Jungermanniales) mitogenome [53]. Repetitive sequences play crucial roles in the structural variability of mitogenomes, as they mediate homologous recombinations, resulting in inversions and translocations [54]. Although empirical evidence indicate that repeats (>50 bp in size; >85% BLASTN identity) can mediate recombination [55], the activity of homologous recombination is directly associated with the length of repeats, where small repeats (50–100 bp) rarely results in recombination [56]. In this context, we have not found any large (>1000 bp) and medium-sized (100–1000 bp) repeats in the *T. atrovirens* mitogenome, except for only nine pairs of small repeats. Our results are in accordance with earlier investigations, suggesting that the structural stability of bryophyte mitogenomes may be attributed in part to the absence of homologous recombination mediated by large repeats [57]. In addition, two other hypotheses have been proposed regarding stability. One is a strengthened nuclear surveillance system [56], and the other one is a large number of polycistronic operons [58]. The four losing genes of *T. atrovirens* mitogenome, namely *matR*, *mttB*, *rps8*, and *rps10*, are also absent in all other mosses [5]. Notably, four ribosomal protein L genes (*rpl2*, *rpl5*, *rpl6*, and *rpl16*) are universally present in the mitogenome of *T. atrovirens* and other species of Dicranales while absent from almost all remaining mosses. 

### 4.3. Phylogenetic Relationships of Pottiaceae

Evolutionary relationships among the three currently recognized bryophyte lineages (hornworts, mosses, liverworts) and tracheophytes have been disputed for a long time, with up to nine competing hypotheses being proposed [59]. Phylogenetic analysis of organelle genomes both indicated that bryophytes were paraphyletic, encompassing three successive sister lineages (hornworts, mosses, and liverworts, in this order). Although the monophyly of bryophytes has been recently proposed by the One Thousand Plant Transcriptomes Initiative [60], our agree with previous studies based on morphology [61] and mitochondrial genomes [5,58], as well as the latest Bryophyte Phylogeny Group (BPG) classification system, which is based on 78 plastid genes under 23 partitioning schemes and three other plastid datasets [62]. Considering that our datasets are comprising distantly-related lineages, codon saturation may cause problems in phylogeny with deep divergence times. We further assessed the saturation (Figure S21) and added the four additional datasets related to “Split codon” to perform the phylogenetic reconstruction. Notably, the phylogeny using the concatenated third codon positions of 15 mitochondrial genes places the hornworts as the earliest divergence within bryophytes, which is consistent with the results of Li et al. (2024) using the amino acid (AA) sequences and the removed third codon site from the nucleotide data [62]. These topological incongruences are likely attributable to biological factors, systematic variations, stochasticity, and treatment errors, which deserve further thorough exploration [63,64]. The phylogeny and classification of the Pottiaceae family have long been controversial [7,8,65]. We reviewed the knowledge on different treatments of the nine genera that are traditionally considered to belong to this family and that are included in this study (Table S21). It is noteworthy that *T. anomala* was located at the base of the order Dicranales, consistent with the recently erected family Timmiellaceae using sequences of *rps4* and *rbcL* genes [66]. Furthermore, the remaining eight Pottiaceae species form a monophyletic group, and agree with the classification of the family into four subfamilies [67]. Specifically, *Scopelophila cataractae* belongs to Merceyoideae and was determined to be the most basal lineage. *Hyophila propagulifera* and *Weissia exserta*, members of Trichostomoideae, are resolved as sister lineages, which are in turn sister to *Streblotrichum convolutum* (Streblotrichoideae). *Barbula unguiculata*, *Pseudocrossidium replicatum*, *S. filaris*, and *T. atrovirens* formed a well-supported clade currently considered Pottioideae. 

### 4.4. Potential DNA Barcoding Regions for Pottiaceae

The classification of Pottiaceae is one of the most challenging tasks of mosses due to factors such as their small plant size, variable morphological characteristics, ambiguous taxonomic significance of several morphological traits, and extensive diversity within some genera [6,7]. Molecular markers have been extensively employed for taxonomic clarification [68] and, recently, several DNA barcodes (e.g., ITS, *matK*, *rbcL*, *rps4*, *trnL*, and *trnH*–*psbA*) are available for bryophytes [69,70]. However, these universal markers have constraints for identifying closely related species within Pottiaceae. In this study, we detected four hypervariable regions (*ndhA*-*ndhH*, *ycf1*-c*hIN*, *ndhF*, and *ndhI*-*ndhA)* thanks to a comprehensive analysis of sequence divergence and nucleotide variability in eight Pottiaceae chloroplast genomes. Indeed, some of these regions have already proven useful, either for other bryophytes (the *ndhH* gene was used to determine species within the genus *Calypogeia* of liverworts [71]) or for plants in general (the *ycf1* gene has been indicated as core DNA barcoding for terrestrial plants [72]). The findings presented here may serve as valuable genetic resources to develop specific Pottiaceae DNA barcodes for conducting systematic and taxonomic studies.

## 5. Conclusions

In this study, we characterized the first-reported organelle genome of the genus *Tortula* and performed a comprehensive analysis among different species of Pottiaceae. *T. atrovirens* has the second-largest chloroplast genome among the eight Pottiaceae species studied herein due to the expansion of IR boundaries. The sizes of mitogenomes of *T. atrovirens* and other mosses showed a narrow range of variation and were smaller than those of hornworts, liverworts, and tracheophytes. Only nine pairs of small repeats were detected, indicating the structural stability of mitogenome may be attributed in part to the absence of homologous recombination-mediated by large repeats. Mapping RNA-seq data to the chloroplast and mitochondrial genome has allowed the detection of a total of six and 51 RNA editing sites, respectively. Further analysis demonstrated that gene content and order of Pottiaceae were highly conserved, thus exhibiting conserved evolution in the organelle genomes. Phylogenetic analysis showed that bryophytes were paraphyletic rather than monophyletic. Furthermore, our results are compatible with the newly erected family Timmiellaceae (from the Pottiaceae family) and confirm that *T. atrovirens* is closely related to *S. filaris* within the Pottioideae subfamily. Additionally, we detected four divergence hotspot regions (*ndhA*-*ndhH*, *ycf1*-*chIN*, *ndhF*, and *ndhI*-*ndhA*) that would serve as valuable genetic resources for candidate-specific DNA barcodes for Pottiaceae. Our research was driven by the imperative to expand the genetic resources available in *Tortula*, thereby contributing to a broader understanding of the evolution of Pottiaceae and the biodiversity conservation of bryophytes. 

## Data Availability

The data presented in this study are openly available in GenBank repository, including the chloroplast genome (accession numbers: PP190927.1) and mitochondrial genome (accession numbers: PP212810.1) of *T. atrovirens*.

## Supplementary Materials

The following supporting information can be downloaded at: https://www.mdpi.com/article/10.3390/genes15060782/s1, Figure S1. Heatmaps of chloroplast (A) and mitochondrial (B) protein-coding genes among 31 species; Figure S2. Sliding window analysis of eight Pottiaceae chloroplast genomes; Figure S3. Codon usage bias of chloroplast (left) and mitochondrial (right) genome in *T. atrovirens*; Figure S4. Schematic diagram of the mitochondrial plastid sequences (MTPTs) in *T. atrovirens*; Figure S5. Synteny analysis of the chloroplast (A) and mitochondrial (B) genome among the eight Pottiaceae species; Figure S6. Heatmaps of pairwise *Ka*/*Ks* values among each shared chloroplast (A) and mitochondrial (B) PCGs of the eight Pottiaceae species; Figure S7. Phylogeny of 31 species based on the concatenated first and second codon positions of 62 chloroplast genes; Figure S8. Phylogeny of 31 species based on the concatenated first and second codon positions of 15 mitochondrial genes; Figure S9. Phylogeny of 31 species based on the concatenated third codon positions of 62 chloroplast genes; Figure S10. Phylogeny of 31 species based on the concatenated third codon positions of 15 mitochondrial genes; Figure S11. Saturation analysis of 62 chloroplast genes under 31 partitioning schemes (left) and 62 chloroplast genes under 31 partitioning schemes (right); Table S1. The best-fitting models of substitution for different partitioning schemes. Table S2. Genomic features of the *T. atrovirens* organelle genome. Table S3. Gene annotation of the *T. atrovirens* chloroplast genome. Table S4. Gene annotation of the *T. atrovirens* mitogenome. Table S5. Dispersed repeats identified in the *T. atrovirens* chloroplast genome. Table S6. Dispersed repeats identified in the *T. atrovirens* mitogenome. Table S7. Tandem repeats identified in the *T. atrovirens* chloroplast genome. Table S8. Tandem repeats identified in the *T. atrovirens* mitogenome. Table S9. SSRs identified in the *T. atrovirens* chloroplast genome. Table S10. SSRs identified in the *T. atrovirens* mitogenome. Table S11. Homologous recombination identified in the *T. atrovirens* mitogenome. Table S12. Relative synonymous codon usage (RSCU) of the *T. atrovirens* chloroplast genome. Table S13. Relative synonymous codon usage (RSCU) of the *T. atrovirens* mitogenome. Table S14. RNA editing sites predicted in PCGs of the *T. atrovirens* chloroplast genome. Table S15. RNA editing sites predicted in PCGs of the *T. atrovirens* mitogenome. Table S16. The homologous DNA fragments identified between the mitogenome and chloroplast genome of *T. atrovirens*. Table S17. The homologous DNA fragments identified between the mitogenome of *T. atrovirens* and the chloroplast genome of *T. lepidozioides*. Table S18. Selective pressure was calculated in the shared PCGs between the Pottiaceae chloroplast genomes. Table S19. Selective pressure calculated in the shared PCGs between the Pottiaceae mitogenomes. Table S20. Species used in Pottiaceae phylogenetic analysis. Table S21. Phylogenetic position of eight genera (Pottiaceae) through history.
