# Supplementary material for: Complete Organelle Genome of the Desiccation-Tolerant (DT) Moss Tortula atrovirens and Comparative Analysis of the Pottiaceae Family
Source: Genes (Basel). 2024 Jun 13;15(6):782. doi: 10.3390/genes15060782 (PMC11202921; doi:10.3390/genes15060782)
Supplement: Supplementary file 1 [file genes-15-00782-s001.zip › Supplementary/Supplementary File.pdf]

SUPPLEMENTARY FIGURES

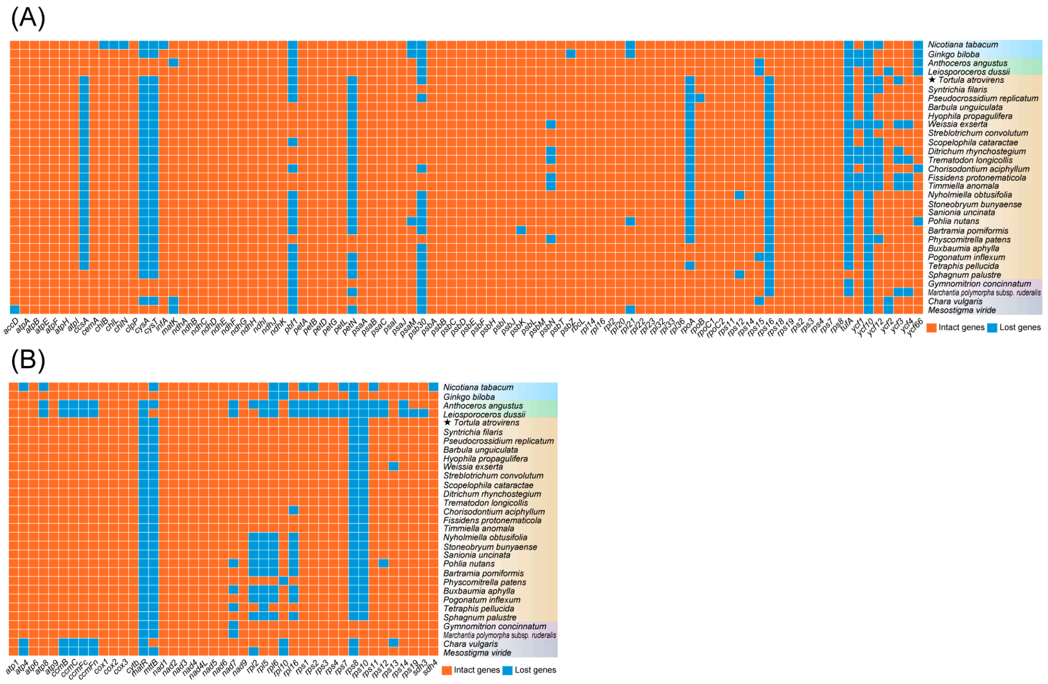

Figure S1. Heatmaps of chloroplast (A) and mitochondrial (B) protein-coding genes among 31 species. Background colors in blue, green, yellow, purple and gray indicate tracheophytes, hornworts, mosses, liverworts, and algaes, respectively. Stars (★) indicate the newly sequenced *T. atrovirens*.

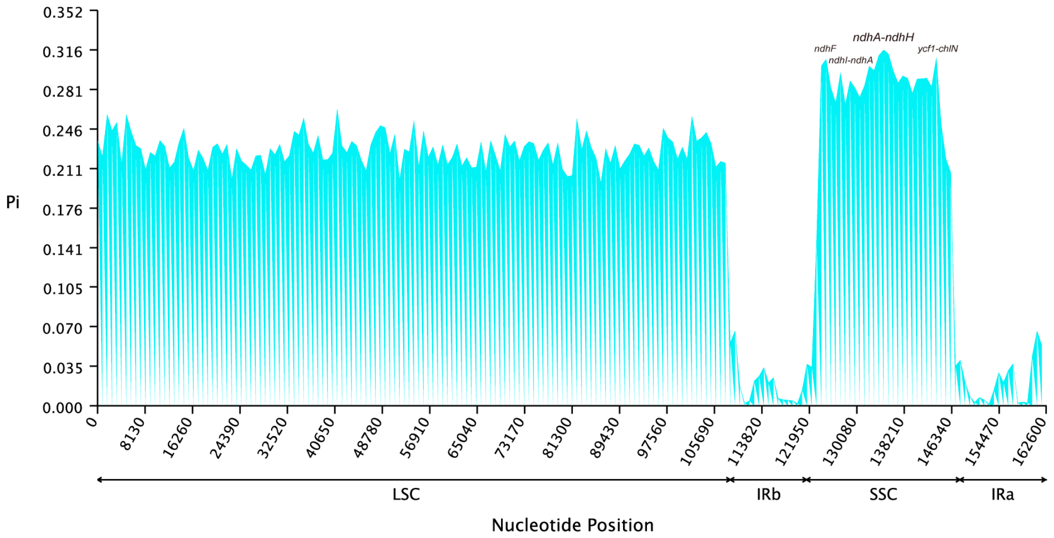

Figure S2. Sliding window analysis of eight Pottiaceae chloroplast genomes.

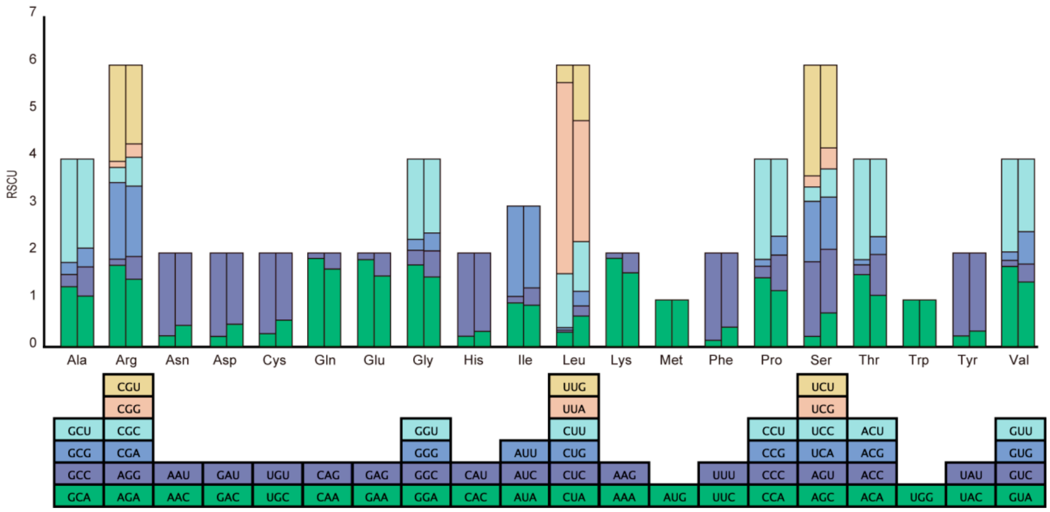

Figure S3. Codon usage bias of chloroplast (left) and mitochondrial (right) genome in *T. atroviren*.

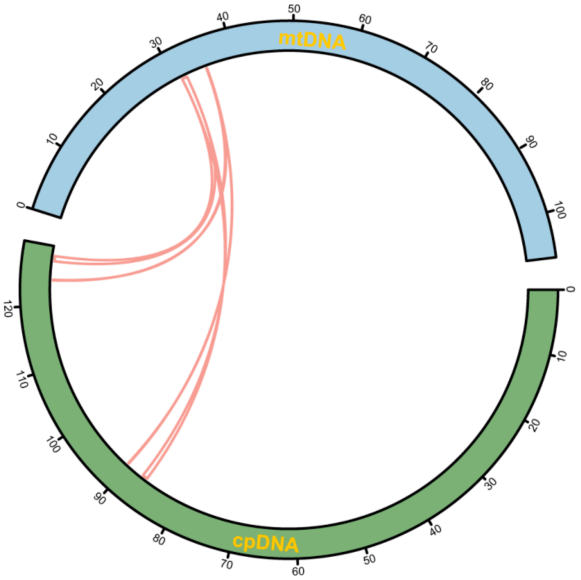

Figure S4. Schematic diagram of the mitochondrial plastid sequences (MTPTs) in *T. atroviren*.

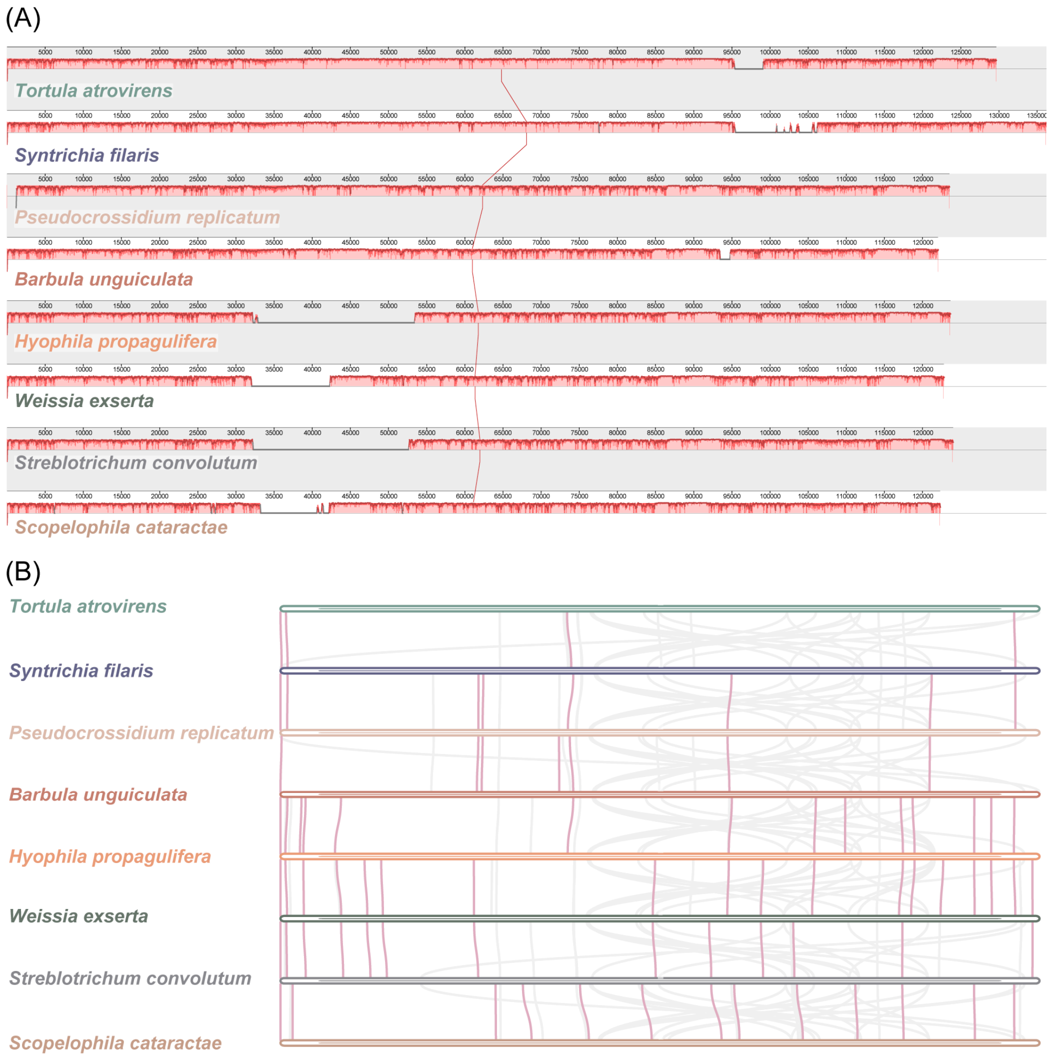

Figure S5. Synteny analysis of the chloroplast (A) and mitochondrial (B) genome among the eight Pottiaceae species.

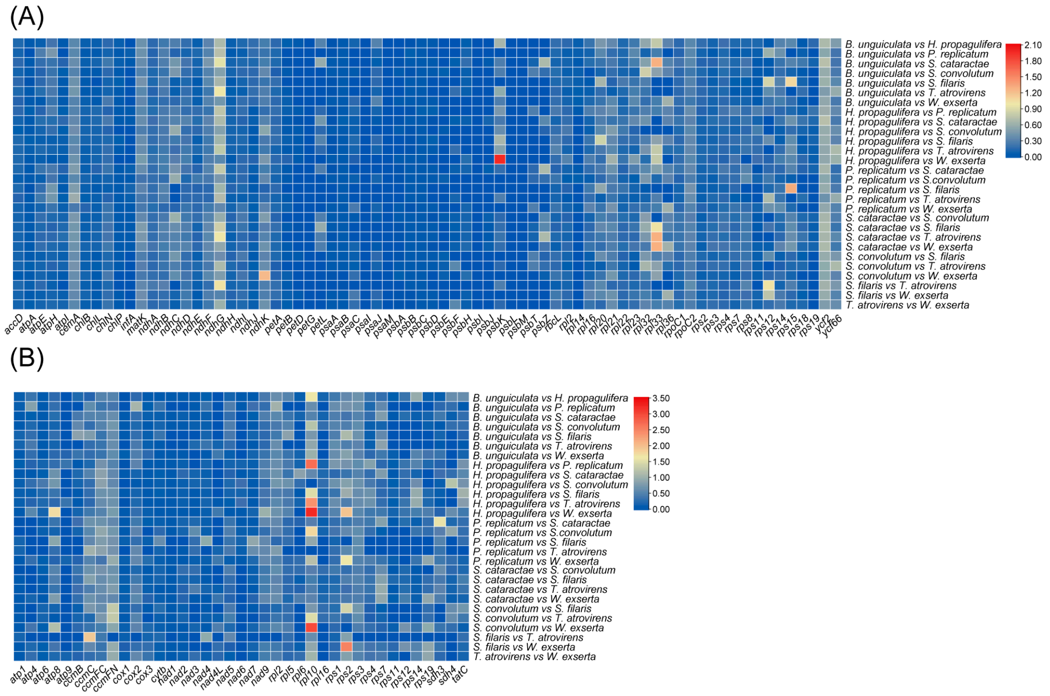

Figure S6. Heatmaps of pairwise *Ka*/*Ks* values among each shared chloroplast (A) and mitochondrial (B) PCGs of the eight Pottiaceae species.

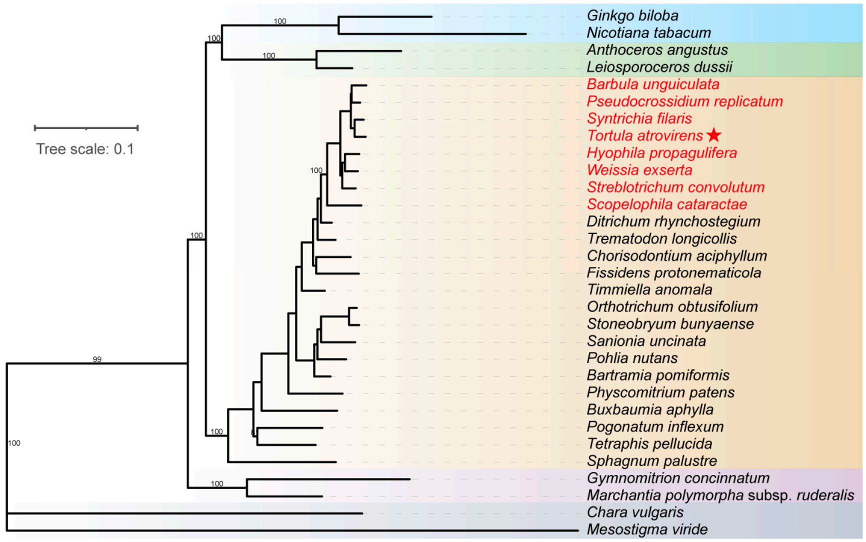

Figure S7. Phylogeny of 31 species based on the concatenated first and second codon positions of 62 chloroplast genes.

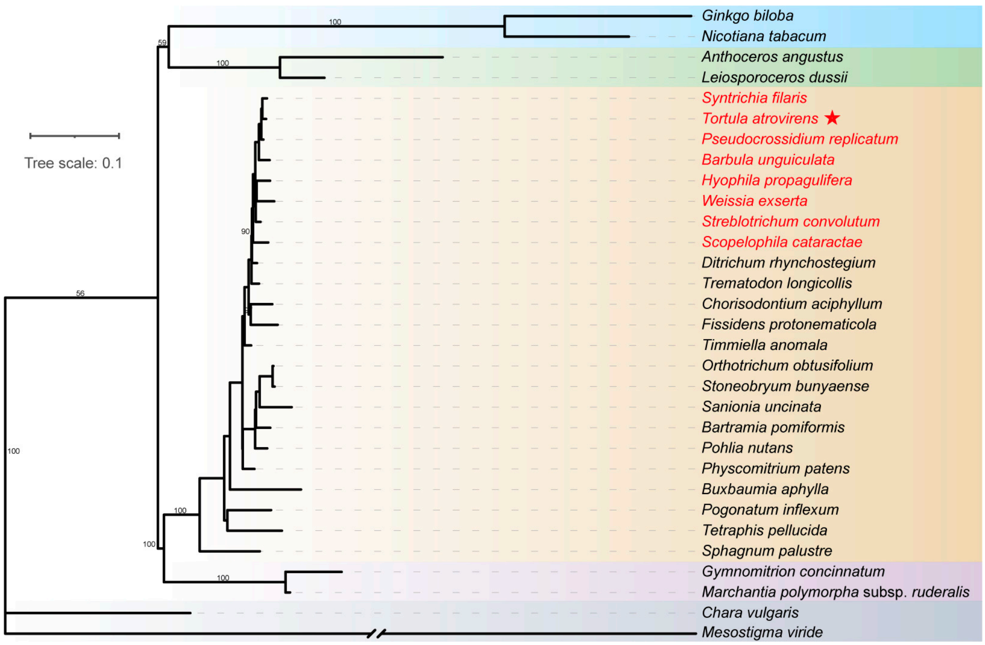

Figure S8. Phylogeny of 31 species based on the concatenated first and second codon positions of 15 mitochondrial genes.

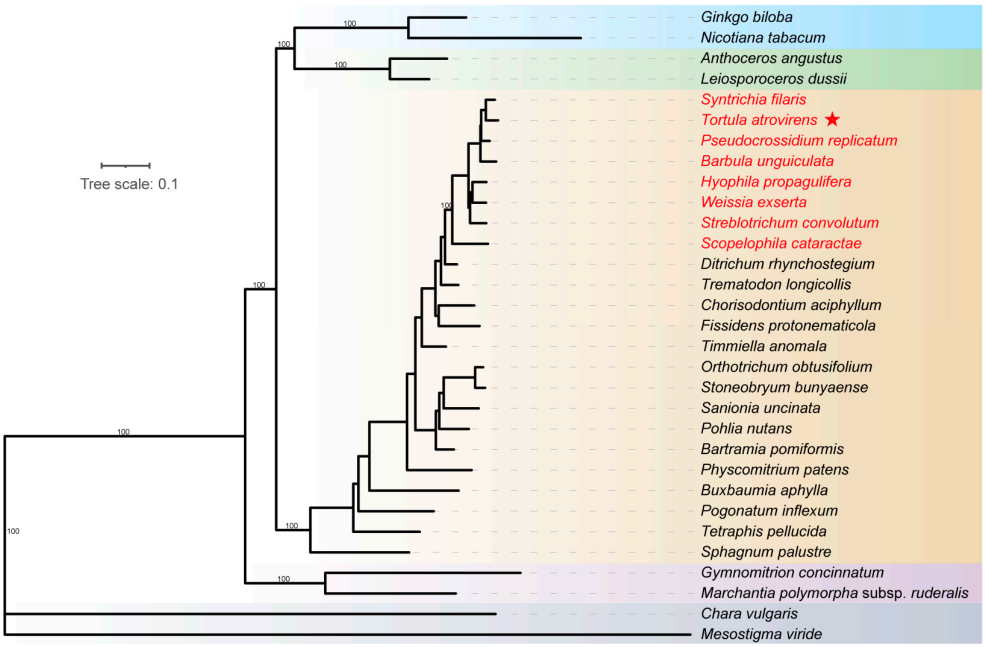

Figure S9. Phylogeny of 31 species based on the concatenated third codon positions of 62 chloroplast genes.

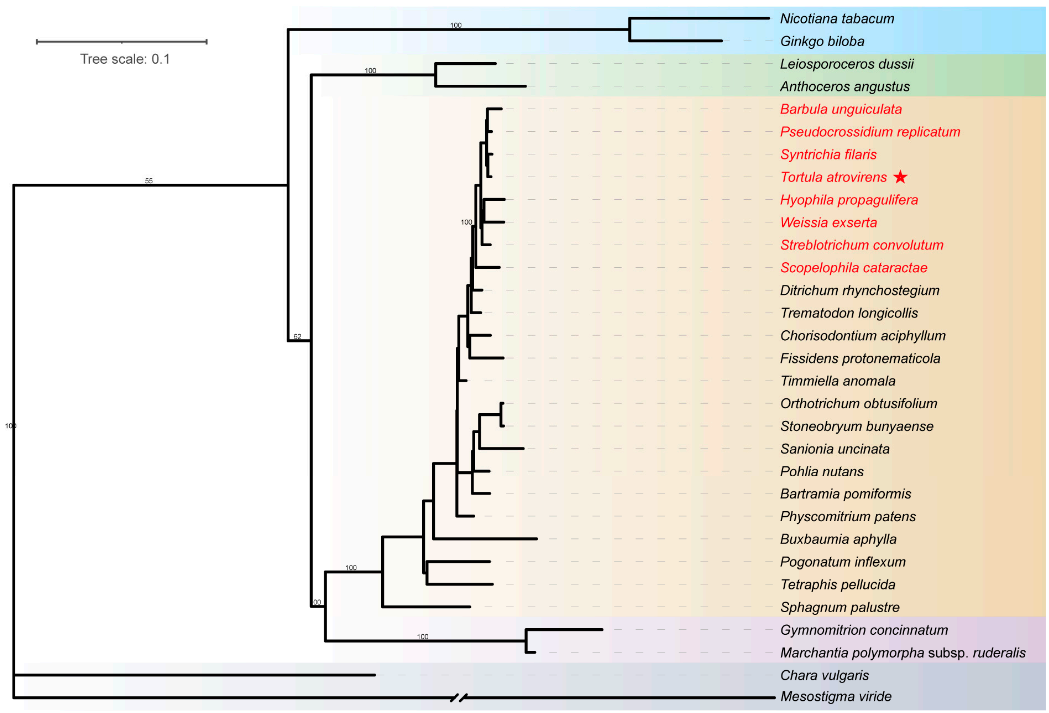

Figure S10. Phylogeny of 31 species based on the concatenated third codon positions of 15 mitochondrial genes.

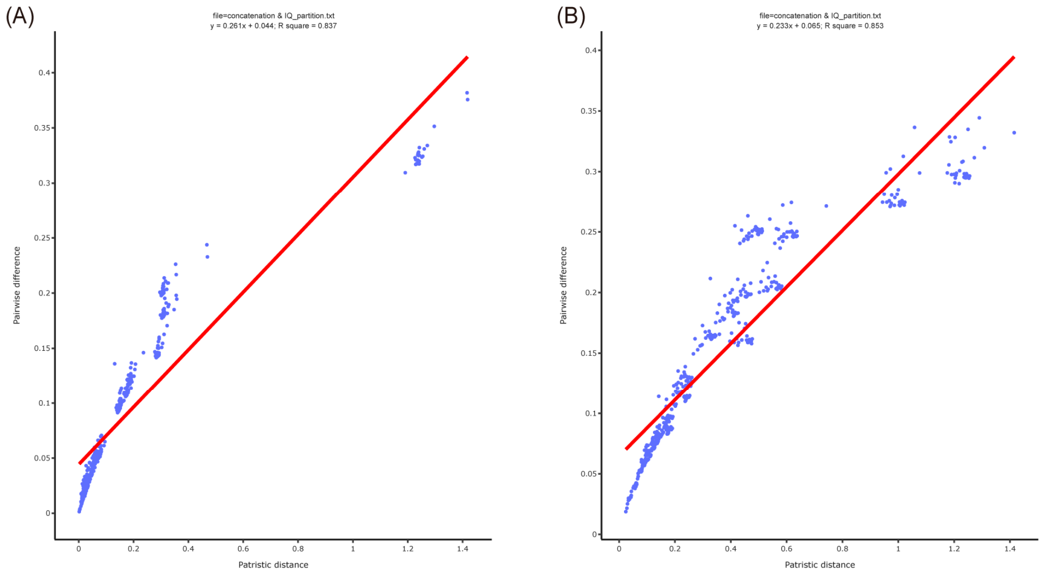

Figure S11. Saturation analysis of 62 chloroplast genes under 31 partitioning schemes (left) and 62 chloroplast genes under 31 partitioning schemes (right).

SUPPLEMENTARY TABLES

Please see Table S1-Table S21 at Excel worksheet.

Table S1. The best-fitting models of substitution for different partitioning schemes.
Table S2. Genomic features of the *T. atrovirens* organelle genome.
Table S3. Gene annotation of the *T. atrovirens* chloroplast genome.
Table S4. Gene annotation of the *T. atrovirens* mitogenome.
Table S5. Dispersed repeats identified in the *T. atrovirens* chloroplast genome.
Table S6. Dispersed repeats identified in the *T. atrovirens* mitogenome.
Table S7. Tandem repeats identified in the *T. atrovirens* chloroplast genome.
Table S8. Tandem repeats identified in the *T. atrovirens* mitogenome.
Table S9. SSRs identified in the *T. atrovirens* chloroplast genome.
Table S10. SSRs identified in the *T. atrovirens* mitogenome.
Table S11. Homologous recombination identified in the *T. atrovirens* mitogenome.
Table S12. Relative synonymous codon usage (RSCU) of the *T. atrovirens* chloroplast genome.
Table S13. Relative synonymous codon usage (RSCU) of the *T. atrovirens* mitogenome.
Table S14. RNA editing sites predicted in PCGs of the *T. atrovirens* chloroplast genome.
Table S15. RNA editing sites predicted in PCGs of the *T. atrovirens* mitogenome.
Table S16. The homologous DNA fragments identified between the mitogenome and chloroplast genome of *T. atrovirens*.
Table S17. The homologous DNA fragments identified between the mitogenome of *T. atrovirens* and the chloroplast genome of *Takakia lepidozioides*.
Table S18. Selective pressure calculated in the shared PCGs between the Pottiaceae chloroplast genomes.
Table S19. Selective pressure calculated in the shared PCGs between the Pottiaceae mitogenomes.
Table S20. Species used in Pottiaceae phylogenetic analysis.
Table S21. Phylogenetic position of eight genus (Pottiaceae) through history.
